# Supplementary material for: Obesity Impairs Cognitive Function with No Effects on Anxiety-like Behaviour in Zebrafish
Source: Int J Mol Sci. 2023 Aug 1;24(15):12316. doi: 10.3390/ijms241512316 (PMC10419065; doi:10.3390/ijms241512316)
Supplement: Supplementary file 1 [file ijms-24-12316-s001.zip › ijms-2526924-supplementary.pdf]

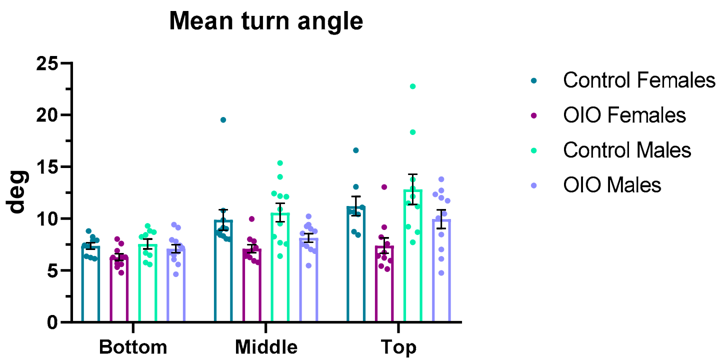

2-way ANOVAs		p-values		
		Feeding ratio	Sex	Interaction
Mean Turn angle	Bottom	0.0553 #	0.2041	0.4262
	Middle	0.0011 **	0.2453	0.8246
	Top	0.0033 **	0.0558 #	0.6589

Figure S1. Mean turn angle in the bottom, middle and top area during NDTD. Significant differences induced by OIO are found in the middle and top zones, but no influence of sex. Data are represented by mean ±SEM and analysed by two-way ANOVA. Asterisks indicate significant differences (** $p \leq 0.01$), and hashtag indicates an apparent significant difference (# $p = 0.051$-0.06).

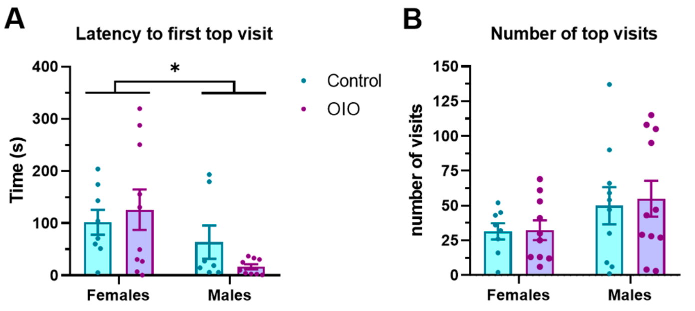

2-way ANOVAs	p-values		
	Feeding ratio	Sex	Interaction
Latency to 1st visit	0.693	**0.0164** *	0.2265
N. top visits	0.7931	0.0704	0.8492

Figure S2. Anxiety-like parameters in the top area after NTDT. (A) Latency to the zone. (B) Number of visits to the top area. Data are represented by mean ±SEM and analysed by two-way ANOVA, asterisks indicate significant differences (*$p \leq 0.05$).

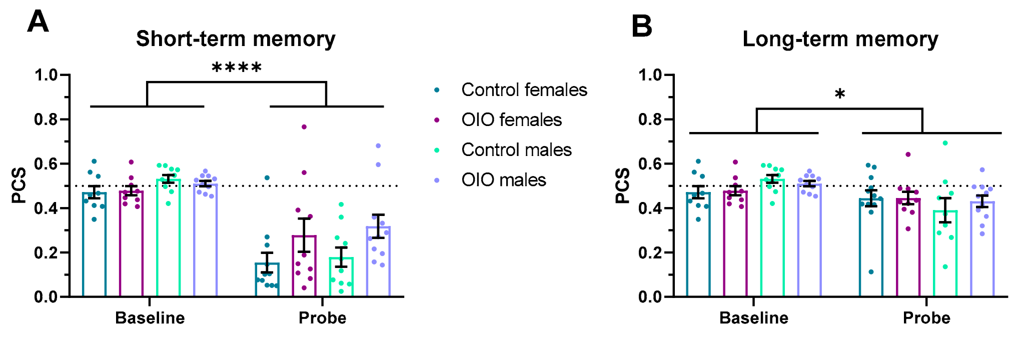

Mixed-effects model	p-values		
STM test	Phase	Sex	Interaction
Control	<0.0001****	0.2439	0.6247
DIO	<0.0001****	0.685	0.6021
LTM test	Phase	Sex	Interaction
Control	0.0257*	0.9229	0.1244
DIO	0.0247*	0.7123	0.3114

Figure S3. Effects of gender on STM and LTM in control and OIO fish. (A) Short-term memory test and (B) long-term memory test. Data are represented by mean ± SEM. The dotted line indicates no stimulus preference (PCS = 0.5), low and high PCS mean less or more time spent in conditioned stimulus (CS), respectively. Results were analysed by Repeated Measures Mixed-effect model; asterisks indicate significant differences between phases, baselines and probes (*$p \leq 0.05$, **** $p \leq 0.0001$).

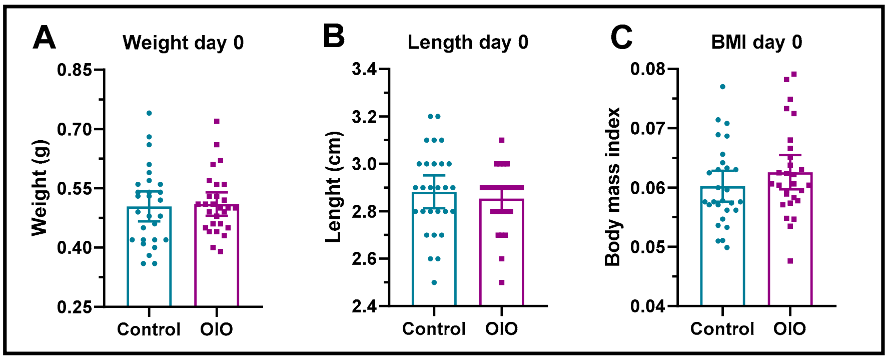

Figure S4. Morphological parameters of control and OIO groups at the beginning of the experiment. (A) Weight (B) Length and (C) Body mass index (BMI). Data are expressed by mean ±SEM analysed by unpaired t-test.
